# Development and psychometric evaluation of item banks for memory and attention – supplements to the EORTC CAT Core instrument

**DOI:** 10.1186/s12955-023-02199-7

**Published:** 2023-11-15

**Authors:** AA Rogge, MA Petersen, NK Aaronson, T Conroy, L Dirven, F Fischer, EJJ Habets, JC Reijneveld, M Rose, C Sleurs, M Taphoorn, KA Tomaszewski, H Vachon, T Young, M Groenvold, on behalf of the EORTC Quality of Life Group

**Affiliations:** 1 Charité Center for Patient-Centered Outcomes Research, Department of Psychosomatic Medicine, Center for Internal Medicine and Dermatology, Charité - Universitätsmedizin Berlin, Berlin, Germany; 2 CPCOR - Charité Center for patient-centered Outcomes Research, Charité - Universitätsmedizin Berlin, Berlin, Germany; 3 Palliative Care Research Unit, Department of Geriatrics and Palliative Medicine GP, Bispebjerg & Frederiksberg Hospital, University of Copenhagen, Bispebjerg bakke 23B, Copenhagen, Denmark 2400; 4 Division of Psychosocial Research & Epidemiology, The Netherlands Cancer Institute, Amsterdam, The Netherlands; 5 Medical Oncology Department, Institut de Cancérologie de Lorraine, Vandoeuvre-lès- Nancy, F-54519 France; 6 Université de Lorraine, APEMAC, équipe MICS, Nancy, F-54000 France; 7 Department of Neurology, Leiden University Medical Center, Leiden, The Netherlands; 8 Department of Neurology, Haaglanden Medical Center, PO Box 432, The Hague, 2501 CK The Netherlands; 9 Department of Medical Psychology, Haaglanden Medical Center, PO Box 432, The Hague, 2501 CK The Netherlands; 10 Department of Neurology & Brain Tumor Center Amsterdam, location VUmc of Amsterdam University Medical Centers, Amsterdam, The Netherlands; 11 Department of Neurology, Stichting Epilepsie Instellingen Nederland (SEIN), Heemstede, the Netherlands; 12 Department of Cognitive Neuropsychology, Tilburg University, Tilburg, Belgium; 13 Department of Oncology, KU Leuven, Leuven, Belgium; 14 Faculty of Medicine and Health Sciences, Andrzej Frycz Modrzewski Kraḱów University, Kraków, Poland; 15 Center for Contextual Psychiatry, Department of Neurosciences, KU Leuven, Leuven, Belgium; 16 Supportive Oncology Research Team, East & North Hertfordshire NHS Trust including Mount Vernon Cancer Centre, Northwood, UK; 17 Department of Public Health, University of Copenhagen, Copenhagen, Denmark

**Keywords:** Cancer, Cognitive functioning, Computerized adaptive testing, EORTC QLQ-C30, Item bank, Self-report

## Abstract

**Background:**

Cancer patients may experience a decrease in cognitive functioning before, during and after cancer treatment. So far, the Quality of Life Group of the European Organisation for Research and Treatment of Cancer (EORTC QLG) developed an item bank to assess self-reported memory and attention within a single, cognitive functioning scale (CF) using computerized adaptive testing (EORTC CAT Core CF item bank). However, the distinction between different cognitive functions might be important to assess the patients’ functional status appropriately and to determine treatment impact. To allow for such assessment, the aim of this study was to develop and psychometrically evaluate separate item banks for memory and attention based on the EORTC CAT Core CF item bank.

**Methods:**

In a multistep process including an expert-based content analysis, we assigned 44 items from the EORTC CAT Core CF item bank to the memory or attention domain. Then, we conducted psychometric analyses based on a sample used within the development of the EORTC CAT Core CF item bank. The sample consisted of 1030 cancer patients from Denmark, France, Poland, and the United Kingdom. We evaluated measurement properties of the newly developed item banks using confirmatory factor analysis (CFA) and item response theory model calibration.

**Results:**

Item assignment resulted in 31 memory and 13 attention items. Conducted CFAs suggested good fit to a 1-factor model for each domain and no violations of monotonicity or indications of differential item functioning. Evaluation of CATs for both memory and attention confirmed well-functioning item banks with increased power/reduced sample size requirements (for CATs ≥ 4 items and up to 40% reduction in sample size requirements in comparison to non-CAT format).

**Conclusion:**

Two well-functioning and psychometrically robust item banks for memory and attention were formed from the existing EORTC CAT Core CF item bank. These findings could support further research on self-reported cognitive functioning in cancer patients in clinical trials as well as for real-word-evidence. A more precise assessment of attention and memory deficits in cancer patients will strengthen the evidence on the effects of cancer treatment for different cancer entities, and therefore contribute to shared and informed clinical decision-making.

**Supplementary Information:**

The online version contains supplementary material available at 10.1186/s12955-023-02199-7.

## Background

 Cancer patients may experience a decrease in cognitive functioning (CF) before, during and after cancer treatment [1–3]. This might include impairments in verbal and visual memory, information processing, attention, executive functioning as well as visuospatial or language skills. As described in a review, up to 75% of cancer patients show and/or report such impairment during cancer treatments [4]. Moreover, up to 35% of cancer survivors indicate long-lasting cancer-related cognitive impairment (CRCI) after completion of treatment [4]. CRCI appears to be associated with lower overall quality of life [5, 6]. Identifying such cognitive impairments is important for clinician awareness, patient education and counseling, and potentially for treatment.

Measuring CRCI includes both objective (e.g., neurological assessment; standardized neuropsychological assessment) [7] and subjective assessments (e.g., patient-reported outcome measures (PROMs)) [8]. Objective neuropsychologic test batteries are typically resource intensive. PROMs as subjective assessments can be used more easily. However, patients might not be able to accurately answer questionnaires due to the degree of cognitive impairment [9]. This indicates the strong need of subjective assessments of CRCI allowing for individual adaptation to the patients’ cognitive ability.

The European Organisation for Research and Treatment of Cancer (EORTC) Quality of Life Group (QLG) included a 2-item Cognitive Functioning (CF) scale within the widely used EORTC QLQ-C30, with one item assessing memory and the other attention [10, 11]. To address the need for measures adapting to the individual’s abilities, the EORTC QLG extended and adapted the CF scale from the EORTC QLQ-C30 to a 34-item bank (EORTC CAT Core CF) allowing for computerized adaptive testing (CAT) [12–14].

Providing CATs might reduce potential ceiling effects of PROM assessment and increase measurement precision. This precision is obtained by adapting item selection to each person, hence, presenting only those items to patients that are likely to be relevant to the individual ability while still maintaining direct comparability across individuals and populations. Therefore, CAT use can reduce response burden, as more relevant items are presented to patients and less items need to be answered in order to obtain sufficient information of functioning.

The EORTC CAT Core CF was developed due to the beneficial implications of CATs (e.g., increased measurement precision and flexibility according to the patient’s needs), while retaining conceptual comparability with the validated 2-item EORTC QLQ-C30 CF scale [13]. For this, attention and memory, were assumed under one scale within the domain of cognitive functioning. However, attention and memory, represent different cognitive functions, and might be shown in different underlying symptoms. Attention refers to collecting and selecting information (focus), while (working) memory refers to processing, storing and retrieval of information. Due to these functional differences, in some cases, the ability to distinguish between limitations in attention and memory may be important from a clinical standpoint [15–17], for instance, when reporting possible cancer treatment effects.

Our aim in the current study was to develop and psychometrically evaluate separate item banks for self-reported memory and attention as supplements to the EORTC CAT Core instrument based on the developmental process of the existing EORTC CAT Core CF item bank [12, 13].

## Methods

### Sample

This study was based on data used within the development of the EORTC CAT Core CF item bank [12, 13]. The sample consisted of 1,030 cancer patients from four countries (Denmark (13.4%), France (15.3%), Poland (27.2%), and United Kingdom (44.1%). Characteristics of the sample were: mean age 63y [range 26–97]; 52.6% female; 23.3% breast cancer as the most common cancer diagnosis; 59.7% cancer stage I-II. Local ethics committees of the participating countries approved the study and written informed consent was obtained before study participation [13].

### Study procedure and statistics

In a multistep process, it was determined whether two item banks, one per domain (memory and attention), could be formed from the existing EORTC CAT Core CF item bank [13], hereafter referred to as the previous study to enhance clarity.

All data analyses were conducted using SAS Enterprise Guide 7.1, except the factor analyses, which were conducted using CBID (https.//biostats-shinyr.kumc.edu/CBID/).

#### Step 1: content analysis for memory and attention items

The static EORTC QLQ-C30 questionnaire consists of 2 CF items only. To develop an item bank suitable for CAT use more CF items are needed. During the development of the EORTC CAT Core CF item bank, further 42 items for memory and attention were generated based on a literature search, expert evaluations, and interviews with cancer patients [12] to provide a comprehensive picture of the memory and attention domains. These 42 items were then formulated in the QLQ-C30 item style, including a 4-point Likert-type scale ranging from ‘not at all’ to ‘very much’ and a recall period of one week [12]. In this paper’s study, these 42 new items together with the two established CF items from the EORTC QLQ-C30 formed the initial item pool. The resulting 44 CF items were then evaluated by experts in the field of neurology, (neuro-)psychology, epidemiology and oncology (n = 5) and assigned to either the memory or attention domain. Successful item allocation was reached, when four out of five experts (80% consensus threshold) agreed on the same domain. Items not reaching the described threshold were discussed within the expert group, and then either allocated to one domain only, or dismissed.

#### Step 2: psychometric analysis of items allocated to the memory and attention domain, respectively

Results of step 1 were subsequently evaluated using confirmatory factor analysis for ordinal data to test item allocation to each domain. In case of misfit to a 1-factor model, items were removed from the item pool. For both domains, higher scores per item and in total reflect better functioning in the patient.

The statistical analyses followed the previous study [13]. Reasonable fit for the 1-factor model was assessed using the following criteria: the root mean square error of approximation (RMSEA) < 0.08, the Tucker-Lewis Index (TLI) and the comparative fit index (CFI) each > 0.95, and the standardized root mean square residual (SRMR) < 0.05 for acceptable/good fit [18, 19]. Threshold levels followed procedure introduced by the COSMIN initiative [20].

#### Step 3: item response theory (IRT) model calibration and evaluation

IRT models assume monotonicity (i.e., increasing likelihood for an item response reflecting good memory functioning with increasing memory score). Hence, monotonicity was assessed for each item by inspecting whether the mean item score increased as the overall rest score increased (the sum score of all items except the item in focus) [21]. Additionally, infit and outfit indices were calculated to further detect differences between the model expected and the actual observed responses to each item (acceptable range between 0.7 and 1.3). Mean items residuals with 95% confidence interval (CI) for each scale respectively were evaluated (acceptable range − 0.1 to 0.1) for a good model fit.

A generalized partial credit model (GPCM) was calibrated to each domain item set [22]. In case of potentially inflated slope parameters possibly caused by local dependence of items [23], an item was re-estimated in a separate model excluding items with high correlations (> 0.8). The slope parameter was then fixed at the obtained estimate and the item added to the full model again. Item residual correlations were used to assess local item independence. Acceptable local independence was assumed if correlations were < 0.25 [24].

Analysis for differential item functioning (DIF) was conducted using ordinal logistic regression for age, sex, cancer site, cancer stage, treatment, country, educational level, cohabitation status, and work status. Due to the large sample size and multiple testing, DIF was regarded as significant if p < .001. Although statistically significant, a potential DIF finding may still have only a trivial impact at the domain score level. Such ‘trivial’ DIF would not be a concern. We mainly aimed to detect indications of practically relevant DIF, i.e., DIF which might have a clinically relevant impact at the domain level. As described in a previous study, translation differences could lead to differences at the domain score level [25], and hence, result in biased findings. To evaluate the practical impact of the possible DIF for IRT score estimation, scores based on the standard model were compared with scores from a model allowing for different parameters in the two DIF groups for the DIF item of focus [13, 26]. If the domain scores obtained with the two models were similar, the DIF was considered not to have practical importance. Here, we were particularly focused on differences greater than the median standard error for the domain score estimated [27].

#### Step 4: evaluation of measurement properties of the item banks used in CAT

For each item bank per domain (memory and attention, respectively) simulations based on observed and Monte Carlo simulated data, respectively, were used to mimic the performance of CATs of varying lengths (1,2,3, to x items). All new CATs were initiated with the original CF item, attention or memory, from the EORTC QLQ-C30. The relative validity (RV) of using the newly developed CAT item bank per domain in comparison to the EORTC QLQ-C30 CF item was evaluated using known group comparisons. For the observed data the following groups were compared: disease stage I and II vs. III and IV; working (yes/no); on treatment (yes/no); the EORTC QLQ-C30 sum scores for the 14 other domains (< 33 vs. ≥33 sum scores).

For the simulated data, 1000 simulations were conducted for each subdomain bank. In each simulation two groups of random size between 50 and 250, were sampled and responses to all items simulated. The groups’ true subdomain scores (memory or attention) differed randomly corresponding to an effect size of 0.2–1.2. Hence, the simulated groups were known to differ. For both the observed and simulated data, RV and sample size requirements of the newly developed CATs compared to using the EORTC QLQ-C30 CF items (memory and attention, respectively) were estimated [28].

## Results

### Step 1: content analysis for memory and attention items

Complete expert agreement for the content allocation was reached for 39 items (88.6%). Two items were allocated to one domain by four of the five experts. For 3 items (items: 11, 13, and 14 in Annex 1) further discussions led to the allocation to one primary domain; resulting in 31 items allocated to memory, and 13 items to attention (see Annex 1).

### Step 2: psychometric analysis of items allocated to the memory and attention subdomain, respectively

The CFA showed good fit to a 1-factor model for each domain, supporting the results of the content analysis by the experts (see Table 1). All items showed acceptable correlation values with the respective EORTC QLQ-C30 item (range *r* = .51 − .79 for memory; range *r* = .63 − .79 for attention). In the previous study, 10 items were removed from the EORTC Core CAT CF item bank due to their misfit with the unidimensional 1-factor model for CF. In this study, it was shown that all items including the 10 items previously removed could be used for the formation of separate memory and attention item banks.

**Table 1:** Fit indices for 1-factor models for memory (31 items) and attention (13 items)

Sub-domain	CFI	TLI	RMSEA	SRMR
Memory	0.98	0.97	0.06	0.04
Attention	1.00	1.00	0.06	0.02

*CFI *Comparative Fit Index (>0.95 for good fit)

*TLI *Tucker-Lewis Index (>0.95 for good fit)

*RMSEA *Root mean square error of approximation (<0.08 for good fit)

*SRMR *Standardised Root Mean Residual (>0.05 for good fit)

#### Memory domain

For the memory item bank (31 items), checking for monotonicity resulted in 65 minor decreases in item scores within 500 checks. Only one decrease, for item 23, showed significance (*p* = .05). Hence, no credible violations of monotonicity were detected, and all 31 items were included in the calibration of the IRT model (see Table 2).

**Table 2:** Slope and location parameters of the estimated IRT models for memory and attention

	Memory		Attention	
Item	Slope	Location	Slope	Location
1			1.31	-1.6
2	1.95	-0.73		
3			1.51	-0.96
4	1.80	-1.54		
5	1.77	-1.2		
6	1.74	-1.83		
7	2.06	-1.08		
8	2.24	-1.36		
9	2.41	-1.07		
10	2.28	-1.36		
11	1.81	-0.72		
12	1.43	-1.41		
13	2.3	-1.39		
14	2.54	-1.38		
15			2.47	-1.23
16			1.84	-1.06
17			3.98	-1.42
18	3.29	-0.85		
19			3.83	-0.85
20	1.35	-1.32		
21	1.59	-1.55		
22	2.76	-1.24		
23	0.99	-2.22		
24	2.65	-0.94		
25			3.15	-1.21
26	1.68	-2.21		
27	3.09	-1.07		
28			3.17	-1.18
29			2.85	-1.33
30	2.52	-1.16		
31	1.72	-1.1		
32			2.0	-1.52
33	2.5	-1.54		
34	2.93	-1.18		
35	2.04	-1.44		
36			4.29	-1.38
37	2.93	-1.38		
38			2.13	-1.03
39	2.64	-1.37		
40	1.79	-1.41		
41	1.69	-1.71		
42	2.45	-1.35		
43	1.38	-1.56		
44			3.53	-1.38

Infit (0.94–1.08) and outfit indices (0.71–1.09) showed acceptable ranges. The mean item residuals with 95% confidence interval (CI) across memory score groups (see Annex 2) indicated that most CIs cover the “no-mean difference” line, and none of the CIs were completely outside the range − 0.1 to 0.1. No non-uniform DIF was found for memory. Some significant results from the DIF analysis in the memory item bank were observed for country and age (see Table 3). The practical impact of the possible DIF for IRT score estimation was evaluated for item 12, age < 50 y vs. > 50y, as this seemed to be the strongest indication of DIF for the memory item bank. Scores based on the two types of models (standard model vs. model accounting for possible DIF), correlated > 0.99 and means differed < 0.1 on a 0-100 scale overall in each of the two age DIF groups. Hence, the significant DIF finding seemed to have a trivial impact on score estimation.

**Table 3:** Significant DIF findings for memory and attention

Item	Domain	Differential Item Functioning (DIF)	DIF effect (ß)
3	Attention	Country (Poland vs. rest)	0.99
4	Memory	Country (France + Poland vs. Denmark + United Kingdom)	1.14
5	Memory	Country (Denmark + France vs. +United Kingdom + Poland)	0.64
8	Memory	Country (Denmark + Poland vs. United Kingdom + France)	0.8
10	Memory	Country (Poland vs. rest)	1.02
12	Memory	Age (< 50y vs. ≥ 50y)	1.2
16	Attention	Country (Poland vs. rest)	1.18
25	Attention	Country (Denmark + Poland vs. France + United Kingdom)	0.81
28	Attention	Country (France + Poland vs. Denmark + United Kingdom)	1.05
32	Attention	Country (Denmark vs. France + Poland)	1.3
35	Memory	Country (Poland vs. rest)	0.73
38	Attention	Country (Poland vs. rest)	1.89
40	Memory	Country (Poland vs. rest)	0.92
41	Memory	Country (Denmark + France vs. + United Kingdom + Poland)	0.88

#### Attention domain

Monotonicity checks showed 18 potential decreases within 200 checks. The decreases were minor (< 3 points decrease on a 0-100 points scale) and all were non-significant. Calibrating the IRT model with no fixed parameters resulted in high slopes (3.9-5.0) for items 17, 19, 29 and 36. These items correlated highly with one or more items. To reduce the risk of inflated slopes, the slopes of these four items were estimated separately in models without the highly correlating item. This resulted in slopes ranging from 2.9 to 4.3. The slope parameters were fixed to these values and the four items added to the full model after which estimation of all other parameters was conducted. Infit indices showed acceptable range (0.78–1). Outfit indices ranged from 0.53 to 0.94, with items 17, 19, 29, 36 and 44 showing values < 0.7. The mean item residuals with 95% CI across attention score groups (see Annex 3) were within the expected range for well-fitting items.

In the attention item bank significant DIF results were detected for country (see Table 3). The practical impact of possible DIF for IRT score estimation was evaluated for item 3 of the attention item bank (Poland vs. the rest), as it indicated the strongest DIF. Scores correlated > 0.99 and means differed < 0.1 on a 0-100 scale; this suggests a trivial impact on score estimation.

### Step 4: evaluation of measurement properties of the item banks used in CAT

**Fig. 1:**
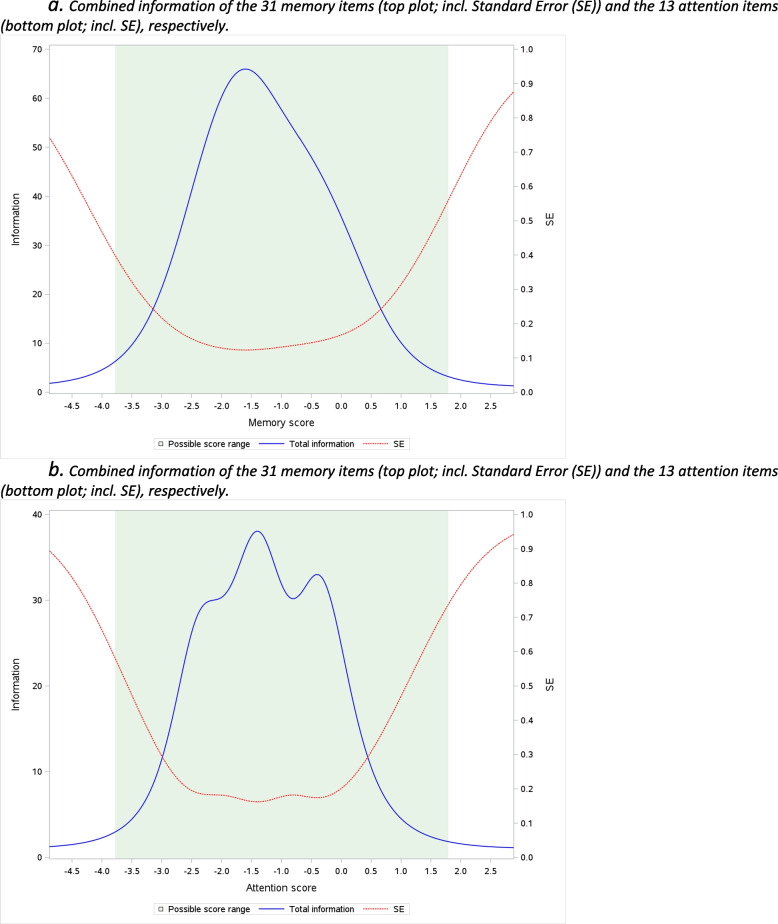
**a** Combined information of the 31 memory items (top plot; incl. Standard Error (SE)) and the 13 attention items (bottom plot; incl. SE), respectively. **b** Combined information of the 31 memory items (top plot; incl. Standard Error (SE)) and the 13 attention items (bottom plot; incl. SE), respectively

 The full memory item bank had a reliability of > 0.9 (corresponding to information > 10) for scores ranging from 3.5 SDs below the sample mean to 1 SD above the mean (i.e., range 4.5 SDs). For attention, the reliability was > 0.9 for a score range of 3.6 SDs. For both item banks, the information functions peaked at scores of approximately 1.5 SD below the sample mean, corresponding to those often reporting ‘quite a bit’ of memory or attention problems (see Fig. 1 for plots of the information functions). The correlation of the two factors was 0.88.

For both the memory and the attention item banks, the CAT simulations showed high correlations between scores of CATs of all lengths (asking 1, 2, 3,… items) and scores of the full item banks. When asking two items, correlations were about 0.90, and using four or more items yielded correlations > 0.95, with < 0.1% of the score estimates deviating > 0.5 SD from the full-scale score.

Evaluations based on both observed and simulated data indicated savings in the relative sample size requirements when using CATs. For the memory item bank, observed and simulated data resulted in similar findings, indicating that when asking only three items, sample size was reduced by 25%. Asking 4–5 items, sample size savings were estimated to be about 30%, increasing to about 35% when asking more than 10 items. For the attention item bank, the observed data simulations indicated sample size requirements may be reduced by 30% when asking ≥ 2 items. The additional savings obtained by increasing the number of items beyond two were modest (< 10%). The simulated data indicated somewhat larger sample size savings of about 40% when asking four items and savings close to 50% when asking ≥ 10 items.

## Discussion

This study investigated whether it is possible to develop two separate item banks for memory as well as attention. Items developed in a rigorous multistep selection procedure [13] were allocated to either the memory or attention domain. The resulting domain-specific item pools were then psychometrically evaluated using CFA as well as calibration of IRT models. The results indicated that two separate item banks could be formed and, after rigorous psychometric evaluation, be used in CAT format.

To our knowledge, this is the first time that two separate item banks for the domains memory and attention have been formed based on the EORTC CAT Core item bank for CF. For this study, the data from as well as the analytic approach employed in a previous psychometric study [13] were used in order to form and evaluate the memory and attention item banks. The new item banks include ten items that were evaluated but not included in the EORTC CAT Core CF item bank due to their misfit with the unidimensional 1-factor.

The availability of separate item banks for memory and attention could be of benefit for at least two scenarios:

First, there is still more research needed to understand the underlying mechanisms of CRCI and its effects on different aspects of CF [3, 29, 30]. The development of domain-specific item banks for CAT assessment might support further in-depth research in the field of CRCI by distinguishing between different functions of cognition (memory vs. attention), offering a more precise measurement via CAT compared to other symptom assessments, and potentially standardizing outcomes for the assessment of cognitive functioning, and symptoms [9].

Secondly, health care professionals need a clear and accurate picture of cancer- and treatment-related symptoms for clinical practice. It is unknown whether treatments affect memory and attention to the same degree and whether the relative impact is the same across treatments. Therefore, as an additional subjective, patient-reported assessment of cognitive problems, item banks developed may aid in understanding the impact of treatments, support information sharing between patient and healthcare provider, and potentially lead to an improved allocation of supportive treatment.

As this study was conducted as an evaluation of concept, limitations for implementation need to be addressed. It is important to determine if these new item banks for memory and attention are responsive to changes in self-reported CF over time. To determine intra-individual change, the newly developed item banks should be used in patients over the course of at least two measurement points [31]. Moreover, clinical studies may evaluate whether the use of the two item banks adds to the understanding of the toxicity of treatments and their temporal course as well as improvement due to interventions on cognitive functioning. Additionally, as subjective and objective CF assessments often show weak to no correlation [32], it would be interesting to investigate on how these new developed item banks might relate to objectively measured CF.

Further research is needed to fully understand CRCI and its correlation with other domains such as emotional functioning and fatigue [33]. The methodological approach described in this paper may be a model to further explore the concept of self-reported CF in cancer patients and its association with other daily life domains.

## Conclusion

This is the first time subdomains were formed from the EORTC CAT Core item bank. We successfully developed and psychometrically evaluated item banks for memory and attention from the well-established EORTC CAT Core item bank for CF [13]. We showed that these separate item banks are feasible and suitable to implement for CAT measurement.

These findings could inform future research on two levels. On a clinical level, providing measures for subdomains of CF such as attention and memory, could support patient-physician communication, for instance, when discussing potential effects of cancer and cancer therapy. This might lead to further improved, and data-driven prediction models on the course of cancer illness. On a methodological level, this study can serve as a blueprint procedure on how to form subdomains from the EORTC CAT Core item bank. For instance, it would be especially interesting to develop separate anxiety and depression subdomains for the existing EORTC CAT emotional functioning domain.

### Supplementary Information

**Additional file 1: Annex 1.** Table 1. Results of content analysis. *items from the EORTC QLQ-C30 for the cognitive functioning domain

**Additional file 2: Annex 2.** Figure 1. Mean item residuals with 95% CI across memory scores for the candidate memory items

**Additional file 3: Annex 3.** Figure 2. Mean item residuals with 95% CI across attention scores for the candidate attention items

## Data Availability

The EORTC CAT Core item banks and any measure based on this are copyrighted, with all rights reserved by the EORTC Quality of Life Group.
