# Supplementary material for: Development and psychometric evaluation of item banks for memory and attention – supplements to the EORTC CAT Core instrument
Source: Health Qual Life Outcomes. 2023 Nov 15;21:124. doi: 10.1186/s12955-023-02199-7 (PMC10647100; doi:10.1186/s12955-023-02199-7)
Supplement: Supplementary file 1 — Additional file 1: Annex 1. Table 1. Results of content analysis. *items from the EORTC QLQ-C30 for the cognitive functioning domain [file 12955_2023_2199_MOESM1_ESM.docx]

Annex 1:

Table 1: Results of content analysis

| *Item* | *Item text* | *Memory* | *Attention* | *EORTC CAT Core* |
| --- | --- | --- | --- | --- |
| *1* | *Have you had difficulty performing two tasks simultaneously, e.g., having a conversation while cooking?* |  | *x* | *X* |
| *2* | *Have you had difficulty remembering words, e.g., a word was “on the tip of your tongue” but you could not quite find it?* | *x* |  |  |
| *3* | *Have you been distracted by thoughts when you should have been concentrating on something else?* |  | *x* | *x* |
| *4* | *Have you had difficulty remembering the names of common things?* | *x* |  |  |
| *5* | *Have you had difficulty remembering what date it was?* | *x* |  | *x* |
| *6* | *Have you had difficulty remembering where things are normally kept?* | *x* |  |  |
| *7* | *Have you had difficulty remembering whether you had already told someone something?* | *x* |  |  |
| *8* | *Have you had difficulty remembering what somebody told you a few minutes earlier?* | *x* |  | *x* |
| *9* | *Have you had difficulty remembering what you were going to say while you were talking?* | *x* |  | *x* |
| *10* | *Have you had difficulty remembering what happened the last few days?* | *x* |  | *x* |
| *11* | *Have you walked into a room but forgotten what you went for?* | *x* |  | *x* |
| *12* | *Have you had difficulty remembering the names of relatives, friends, or other people you see regularly?* | *x* |  | *x* |
| *13* | *Have you had difficulty remembering what you initially were doing if you started to do something else in the meantime?* | *x* |  | *x* |
| *14* | *Have you had difficulty remembering what you were doing when you were interrupted?* | *x* |  | *x* |
| *15* | *Have you had difficulty remembering in concentrating on things, like reading a newspaper or watching television?* |  | *x* | *x (*Q20)* |
| *16* | *Have you been reading something and had to read the same lines again because you were distracted?* |  | *x* | *x* |
| *17* | *Have you had difficulty staying focused on a task or an activity even when it was interesting?* |  | *x* |  |
| *18* | *Have you had difficulty remembering things?* | *x* |  | *X (*Q25)* |
| *19* | *Have you had difficulty maintaining concentration even when something really interested you?* |  | *x* | *x* |
| *20* | *Have you had difficulty remembering where you left things, e.g., your keys or your wallet?* | *x* |  |  |
| *21* | *Have you had difficulty remembering appointments or meetings?* | *x* |  |  |
| *22* | *Have you had difficulty remembering what somebody told you a few days earlier?* | *x* |  |  |
| *23* | *Have you had difficulty recognizing faces of people you have seen before?* | *x* |  |  |
| *24* | *Have you been forgetful?* | *x* |  | *x* |
| *25* | *Have you had difficulty paying attention on a task or a conversation for a longer period of time?* |  | *x* | *x* |
| *26* | *Have you had difficulty recognising relatives, friends, or other people you see regularly?* | *x* |  | *x* |
| *27* | *Have you had difficulty remembering what somebody just told you?* | *x* |  | *x* |
| *28* | *Have you had difficulty paying attention for as long as you wanted or needed?* |  | *x* | *x* |
| *29* | *Have you had difficulty paying attention (e.g., when watching a movie, reading or, talking to someone)?* |  | *x* |  |
| *30* | *Have you had difficulty remembering new information, like a person’s name or simple instructions?* | *x* |  | *x* |
| *31* | *Have you had difficulty remembering to take things you needed with you?* | *x* |  | *x* |
| *32* | *Have you become more distracted from a task before finishing it?* |  | *x* | *x* |
| *33* | *Have you had difficulty remembering whether you had already done something?* | *x* |  | *x* |
| *34* | *Have you had difficulty remembering something you had just said?* | *x* |  | *x* |
| *35* | *Have you had difficulty remembering to pass on a message or remind someone of something?* | *x* |  | *x* |
| *36* | *Have you had difficulty maintaining concentration even when doing something important?* |  | *x* | *x* |
| *37* | *Have you had difficulty remembering what you were just thinking?* | *x* |  | *x* |
| *38* | *Have you had difficulty gathering your thoughts?* |  | *x* | *x* |
| *39* | *Have you had difficulty remembering to do the things you had planned to do?* | *x* |  | *x* |
| *40* | *Have you had difficulty remembering what weekday it was?* | *x* |  | *x* |
| *41* | *Have you had difficulty remembering what a text you were reading was about?* | *x* |  | *x* |
| *42* | *Have you had difficulty remembering what you did a few days earlier?* | *x* |  | *x* |
| *43* | *Have you forgotten to do routine things such as turning off the light or locking the door?* | *x* |  | *x* |
| *44* | *Have you had difficulty staying focused on a task or an activity?* |  | *x* | *x* |

*items from the EORTC QLQ-C30 for the cognitive functioning domain
