# Supplementary figures and images for: Development and psychometric evaluation of item banks for memory and attention – supplements to the EORTC CAT Core instrument
Source: Health Qual Life Outcomes. 2023 Nov 15;21:124. doi: 10.1186/s12955-023-02199-7 (PMC10647100; doi:10.1186/s12955-023-02199-7)

Annex 2:

Figure 1. Mean item residuals with 95% CI across memory scores for the candidate memory items
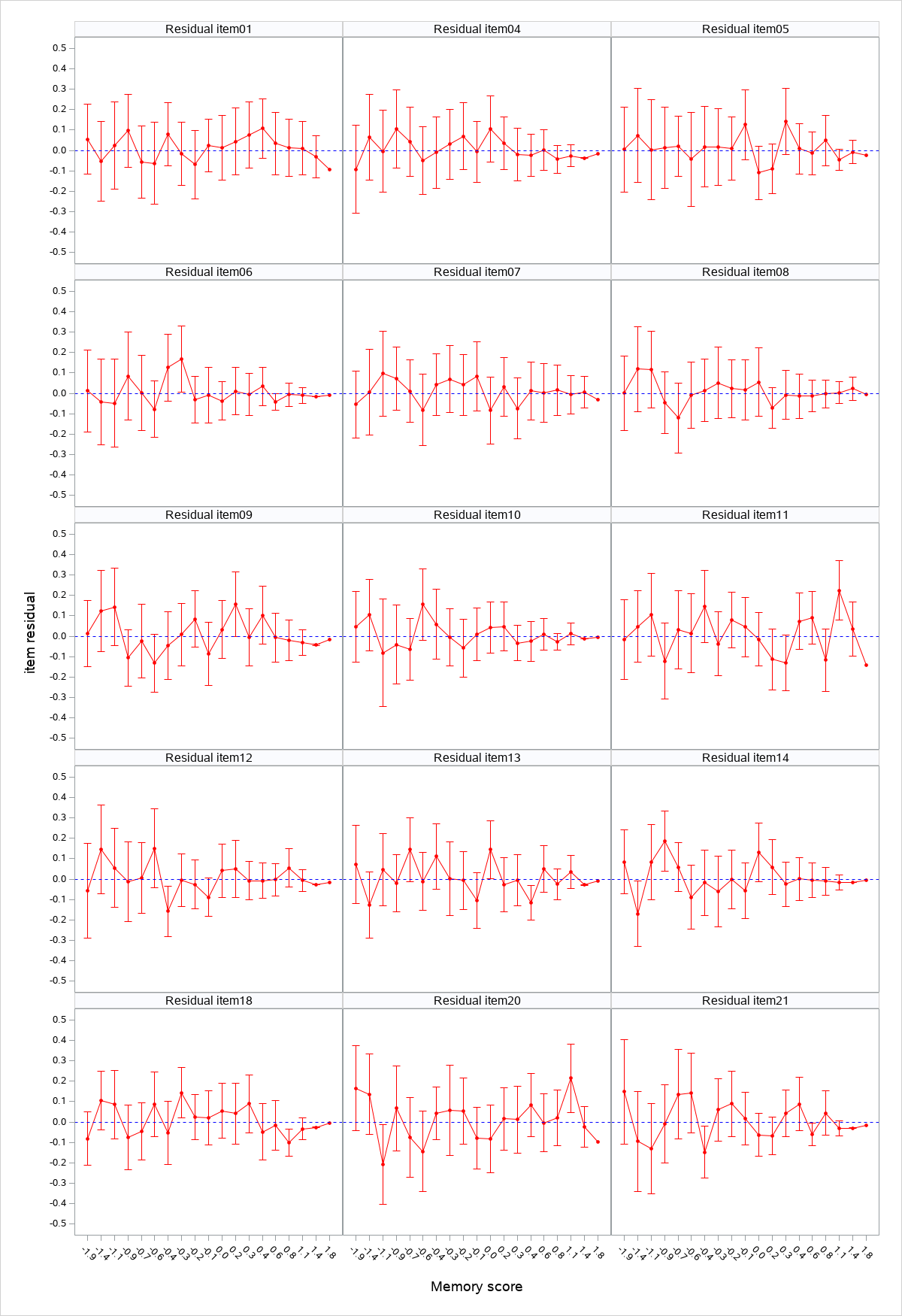


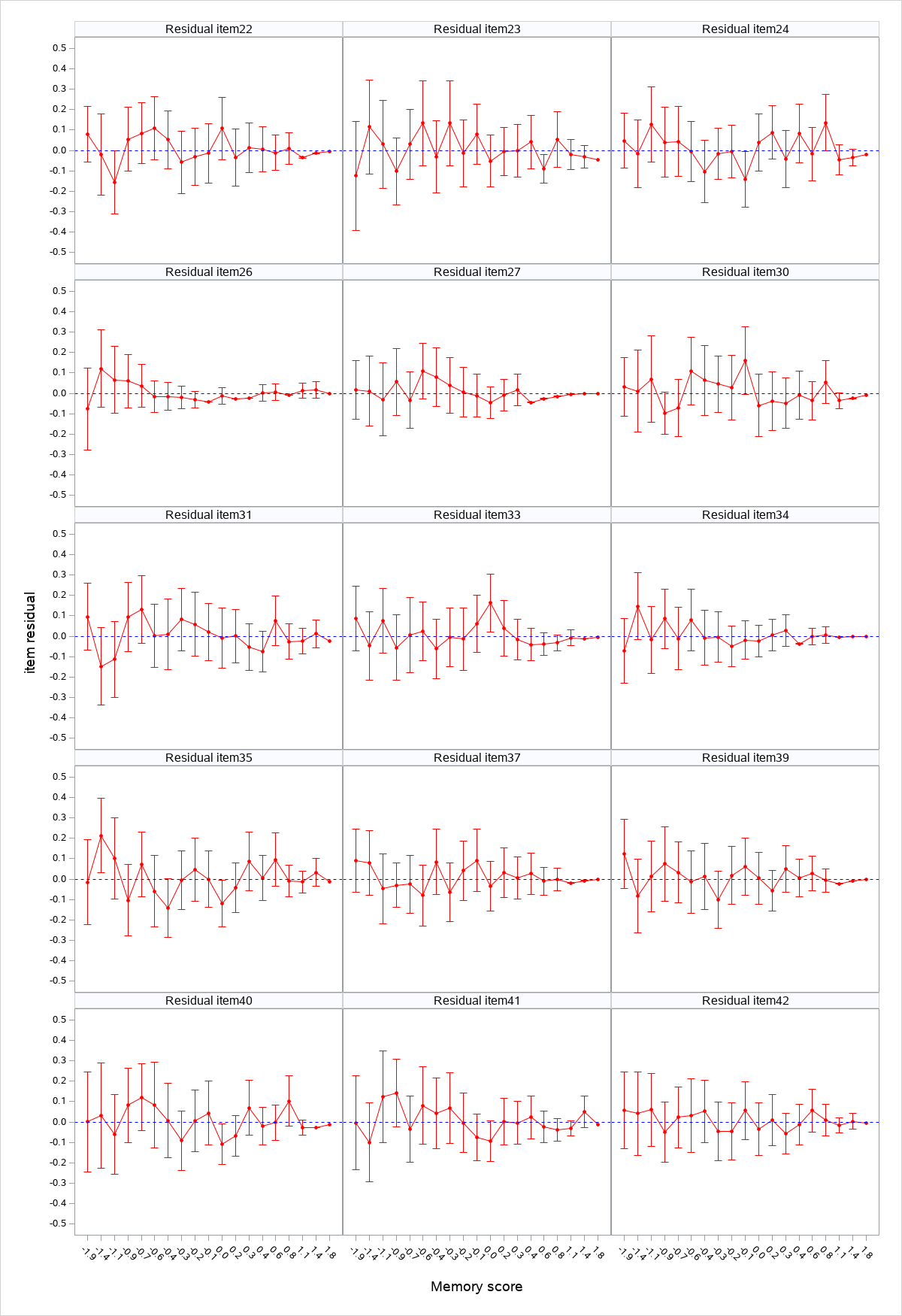


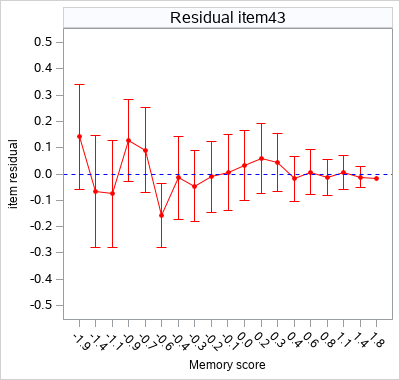

Supplement: Supplementary file 2 — Additional file 2: Annex 2. Figure 1. Mean item residuals with 95% CI across memory scores for the candidate memory items [file 12955_2023_2199_MOESM2_ESM.docx]
