# Supplementary material for: Development and psychometric evaluation of item banks for memory and attention – supplements to the EORTC CAT Core instrument
Source: Health Qual Life Outcomes. 2023 Nov 15;21:124. doi: 10.1186/s12955-023-02199-7 (PMC10647100; doi:10.1186/s12955-023-02199-7)

Annex 3

Figure 2. Mean item residuals with 95% CI across attention scores for the candidate attention items


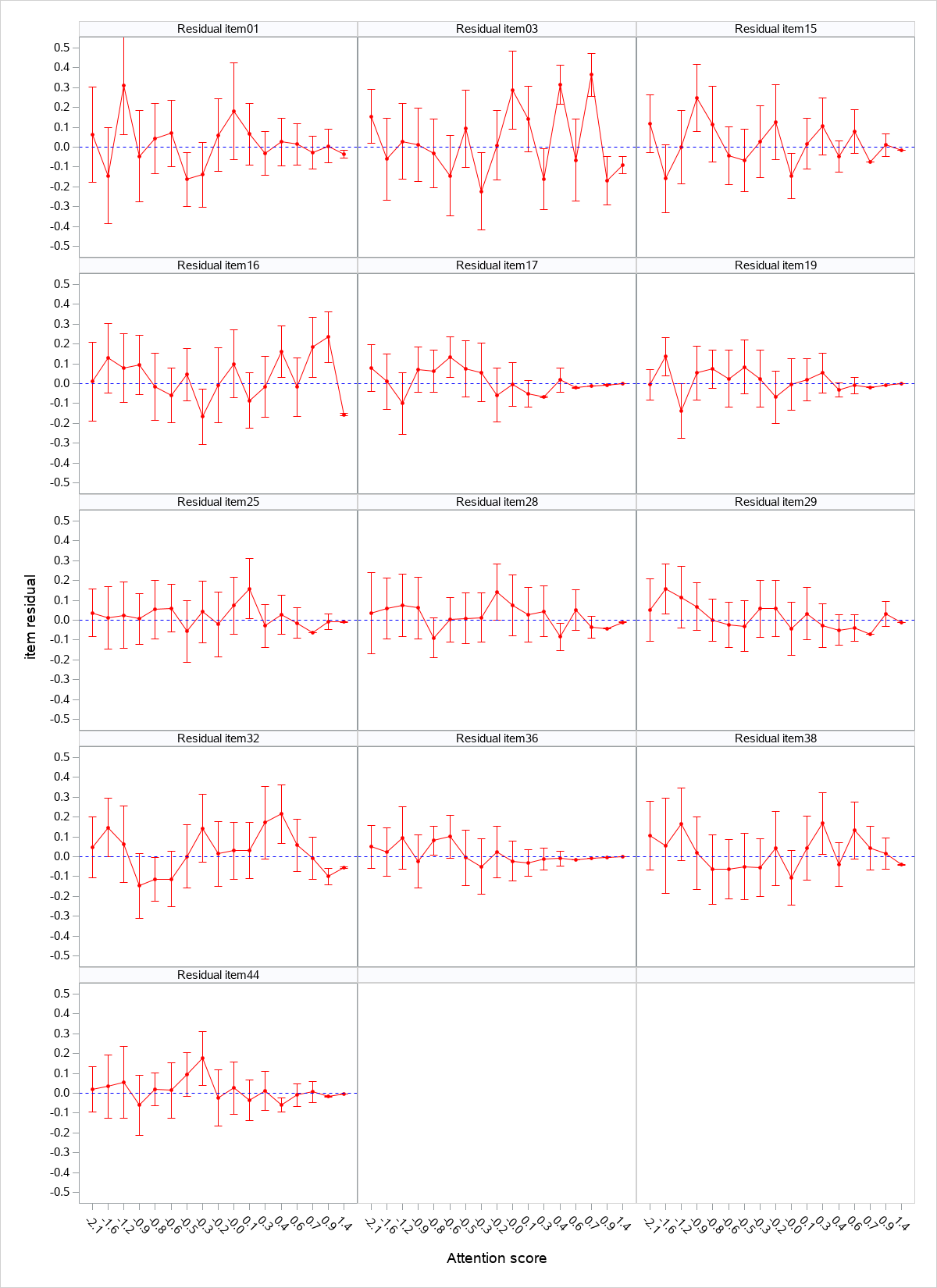

Supplement: Supplementary file 3 — Additional file 3: Annex 3. Figure 2. Mean item residuals with 95% CI across attention scores for the candidate attention items [file 12955_2023_2199_MOESM3_ESM.docx]
